# Editorial: The repair of DNA–protein crosslinks

**DOI:** 10.3389/fmolb.2023.1203479

**Published:** 2023-04-28

**Authors:** Yilun Sun, John L. Nitiss, Yves Pommier

**Affiliations:** ^1^ Laboratory of Molecular Pharmacology, Developmental Therapeutics Branch, Center for Cancer Research, National Cancer Institute, National Institutes of Health, Bethesda, MD, United States; ^2^ Pharmaceutical Sciences Department, University of Illinois College of Pharmacy, Rockford, IL, United States

**Keywords:** DNA-protein crosslink (DPC), topoisomerases (topo), ubiquitin-proteasome degradation, SPRTN protease, sumoylation, endonucleases, FAM111A

DNA–protein crosslinks (DPCs) are among the most ubiquitous and heterogenous lesions that arise from the covalent linking of a protein with a nucleotide residue on DNA (Stingele et al., 2017; Kuhbacher and Duxin, 2020; Weickert and Stingele, 2022). DPCs were initially observed in bacterial and eukaryotic cells irradiated with ultraviolet light (Alexander and Moroson, 1962; Smith, 1962). It was subsequently shown that DPCs could be generated endogenously by certain types of DNA lesions and upon exposure to a variety of physical and chemical agents including aldehydes, metal ions, and ionizing radiation, as well as chemotherapeutics such as topoisomerase inhibitors, DNA methyltransferase inhibitors, and platinum-based drugs (Klages-Mundt and Li, 2017). Recent studies have broadly examined the set of proteins covalently trapped on DNA (Kiianitsa and Maizels, 2020). In addition to proteins that form covalent intermediates during their reactions with DNA, polypeptides that become covalent protein/DNA adducts include abundant proteins such as histones and RNA splicing proteins.

The protein bulk of a DPC imposes steric obstacles on virtually all aspects of the DNA metabolism, including replication, transcription, and remodeling, and hence hampers these activities (Zhang et al., 2020). Therefore, accurate repair of DPCs is the key to genomic DNA fidelity, and failure to repair DPCs has been shown to be implicated in premature aging, carcinogenesis, and the etiology of many other diseases (Fielden et al., 2018; Semlow and Walter, 2021). While DPCs are a significant menace to chromosomal integrity, they have received less attention than the other types of DNA damage, and in consequence, less is understood about how cells repair or tolerate DPCs. Since some DPCs are associated with DNA breaks, their repair requires not only the removal of the bulky protein component but also the repair of the associated broken DNA ends (Sun et al., 2020a). Such an intricacy has been a major hurdle to the elucidation of the overall pathways of DPC repair.

In recent years, several proteases have emerged as parallel/epistatic repair pathways for DPCs by cleaving the protein adducts. The metalloprotease Wss1 (in *S. cerevisiae*)/Dvc-1 (in *C. elegans*)/SPRTN (in metazoans) (Stingele et al., 2014; Lopez-Mosqueda et al., 2016; Stingele et al., 2016; Ruggiano and Ramadan, 2021) and the 26S proteasome (Sun et al., 2020b; Sun et al., 2020c; Sun et al., 2021) are the two most studied proteolytic mechanisms. SPRTN is a replication-coupled protease that digests both break-associated DPC substrates (e.g., topoisomerase DPCs or TOP-DPCs) and DPC substrates without DNA breaks in a sequence-independent manner. Biallelic mutations in SPRTN have been shown to be implicated in Ruijs-Aalfs (RJALS) syndrome, characterized by hepatocellular carcinoma and segmental progeria (Perry and Ghosal, 2022). The 26S proteasome targets DPCs through polyubiquitylation of the substrates throughout the cell cycle. The engagement of the proteasome for DPC repair is dictated by the cellular context where the DPCs are formed (replication, transcription, etc.) (Larsen et al., 2019) and by the ubiquitin E3 ligase that ubiquitylates the DPC substrates (Sun et al., 2020c; Saha et al., 2020). In addition, several other enzymes, including FAM111A and FAM111B (Kojima et al., 2020; Welter and Machida, 2022), DDI1 and DDI2 (Dirac-Svejstrup et al., 2020; Yip et al., 2020), and ACRC/GCNA (Borgermann et al., 2019), have emerged as potential repair proteases for DPCs. Both SPRTN and FAM111A appear to bind nascent DNA and prevent replication fork stalling by removing topoisomerase I (TOP1)-DPCs ahead of the fork and TOP3A-DPCs behind the replication forks (Saha et al., 2023). In addition, SPRTN processes formaldehyde-induced non-specific DPCs, whereas FAM111A targets non-covalent PARP-DNA complexes, suggesting a difference in substrate preference of these proteases. The redundancy of the proteasome complex and proteases such as SPRTN, FAM111, and DDI for replication-associated DPCs remains perplexing. As the expressions of the ubiquitin–proteasome system and the proteases vary across cell lines and tissues, one possibility is that the most expressed protease in a cell line plays the dominant role in DPC repair during replication.

Enzymatic DPCs such as TOP-DPCs are formed through the covalent linkage between the active tyrosine residue of the enzyme and the DNA backbone (Pommier et al., 2016). This covalent complex is a reversible intermediate generated during the normal catalytic reaction of the enzymes but is converted into long-lived DPCs upon exposure to their inhibitors. Following the degradation of TOP-DPCs, the otherwise hidden broken ends are exposed, allowing nucleases specialized for topoisomerase DPCs, tyrosyl-DNA phosphodiesterases 1 and 2, to access and hydrolyze the covalent bond to remove the remaining peptides and liberate the breaks for repair by homologous recombination (HR) or non-homologous end-joining (Pouliot et al., 1999; Ledesma et al., 2009; Sun et al., 2020a; Saha et al., 2023). Topoisomerase II (TOP2)-DPCs with TOP2 proteins covalently bound to the 5′termini ends were found to be repaired by the Mre11/Rad50/Nbs1 (MRN) complex, which cleaves the DNA backbone in the vicinity of the DPCs and releases TOP2 attached to oligonucleotides (Aparicio et al., 2016; Hoa et al., 2016). Recent studies have showed that this process is also used to remove TOP3A-DPC in the late S- and G2-phase (Saha et al., 2023). Our work in this Research Topic shows that the MRN complex can remove TOP2-DPCs by using its endonuclease activity independently of the proteasomal degradation of the DPCs. Surprisingly, the removal of TOP2-DPCs by the MRN complex was found to require the unfolding activity of VCP/p97, suggesting that unfolding of TOP2-DPCs allows the loading of the MRN repair machinery on the DNA fragment adjacent to the DPCs and hence the incision of the DNA (Sun et al., 2022).

Non-enzymatic DPCs are crosslinked by reactive metabolites such as aldehydes to DNA bases without disruption of the phosphodiester bond of the DNA backbone. However, these no-end DPCs were found to accumulate double-strand breaks (DSBs) to activate the HR pathway or translesion synthesis (TLS) (Nakano et al., 2009). Recently, FANCJ a 5′-to-3′ helicase was shown to be required for supporting the bypass of stable DPCs and for the unfolding of the protein adduct (Yaneva et al., 2023). This step likely precedes the degradation of the adduct by SPRTN. These results suggest the unfolding of the protein component may be a common processing step in preventing DPCs from blocking DNA metabolic events and in their eventual repair (Mailand, 2023).

This Research Topic provided both original studies and reviews on DPC repair in mammalian cells, focusing on proteases such as SPRTN and FAM111A and the mechanisms by which they proteolyze DPC substrates (Perry and Ghosal, 2022; Welter and Machida, 2022), post-translational modifications including ubiquitylation, SUMOylation, and ADP-ribosylation in the regulation of DPC repair pathways (Leng and Duxin, 2022), and the nuclease MRN complex and its role in TOP2-DPC repair (Sun et al., 2022). We hope that the original research and reviews presented here will stimulate further studies on some of the major unanswered questions relating to the genesis and repair of DPCs.
